# Influence of Electron Donors on the Charge Transfer Dynamics of Carbon Nanodots in Photocatalytic Systems

**DOI:** 10.1021/acscatal.4c02327

**Published:** 2024-07-26

**Authors:** Stuart Macpherson, Takashi Lawson, Anna Abfalterer, Paolo Andrich, Ava Lage, Erwin Reisner, Tijmen G. Euser, Samuel D. Stranks, Alexander S. Gentleman

**Affiliations:** † Department of Physics, Cavendish Laboratory, University of Cambridge, Cambridge CB3 0HE, U.K.; ‡ Department of Materials Science and Metallurgy, University of Cambridge, Cambridge CB3 0FS, U.K.; § Yusuf Hamied Department of Chemistry, University of Cambridge, Cambridge CB2 1EW, U.K.; ∥ Department of Chemical Engineering and Biotechnology, University of Cambridge, Cambridge CB3 0AS, U.K.

**Keywords:** carbon nanodots, charge transfer, electron donors, laser spectroscopy, photocatalysis, transient absorption

## Abstract

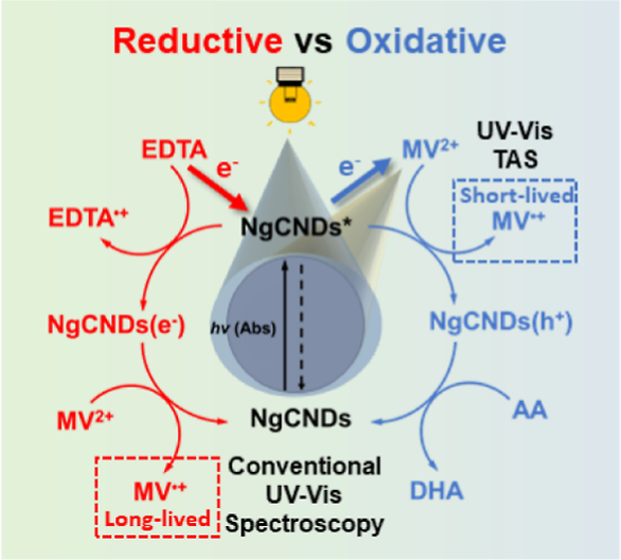

Carbon nanodots (CNDs) are nanosized light-harvesters emerging as next-generation photosensitizers in photocatalytic reactions. Despite their ever-increasing potential applications, the intricacies underlying their photoexcited charge carrier dynamics are yet to be elucidated. In this study, nitrogen-doped graphitic CNDs (NgCNDs) are selectively excited in the presence of methyl viologen (MV^2+^, redox mediator) and different electron donors (EDs), namely ascorbic acid (AA) and ethylenediaminetetraacetic acid (EDTA). The consequent formation of the methyl viologen radical cation (MV^•+^) is investigated, and the excited charge carrier dynamics of the photocatalytic system are understood on a 0.1 ps–1 ms time range, providing spectroscopic evidence of oxidative or reductive quenching mechanisms experienced by optically excited NgCNDs (NgCNDs*) depending on the ED implemented. In the presence of AA, NgCNDs* undergo oxidative quenching by MV^2+^ to form MV^•+^, which is short-lived due to dehydroascorbic acid, a product of photoinduced hole quenching of oxidized NgCNDs. The EDTA-mediated reductive quenching of NgCNDs* is observed to be at least 2 orders of magnitude slower due to screening by EDTA-MV^2+^ complexes, but the MV^•+^ population is stable due to the irreversibly oxidized EDTA preventing a back reaction. In general, our methodology provides a distinct solution with which to study charge transfer dynamics in photocatalytic systems on an extended time range spanning 10 orders of magnitude. This approach generates a mechanistic understanding to select and develop suitable EDs to promote photocatalytic reactions.

## Introduction

Carbon nanodots (CNDs) have materialized as photosensitizers for use in photocatalysis,^1−4^ including H_2_ solar fuel synthesis via H_2_O splitting or solar reforming of biomass waste, alcohol synthesis via CO_2_ reduction with H_2_, or ammonia synthesis via N_2_ reduction with H_2_.^5−9^ Promising features include their favorable optical properties (broadband light absorption and efficient photoluminescence emission) and environmental friendliness (low toxicity, low-energy synthesis, low cost and high water solubility resulting in good biocompatibility), yielding a sustainable light-harvesting component with potential for large-scale production.^10−13^ CND-photocatalyzed systems have displayed excellent activities for H_2_ synthesis (13,450 μmol H_2_ g_CND_^–1^ h^–1^) in conjunction with a Ni bis(diphosphine) cocatalyst (NiP) under simulated solar light (AM 1.5G, 100 mW cm^–2^),^9^ and high turnover frequencies for CO_2_ reduction (3.5 × 10^3^ h^–1^) in conjunction with formate dehydrogenase under simulated solar light.^4^ CNDs have even been employed as photocatalysts to drive organic transformations, such as fluoroalkylation and epoxide ring-opening.^5,8,14^ As such, further improvements in performance through mechanistic understanding are desirable for CNDs to reach their full range of applications.

In general, significant activity enhancement is best pursued via “bottom-up” characterization that elucidates mechanisms governing the various reaction rates relevant to processes underlying the photocatalytic cycle. For instance, dynamic studies of CNDs have utilized time-resolved photoluminescence (PL) spectroscopy^15−17^ and transient absorption (TA) spectroscopy^18^ to gain insight into the optoelectronic excited state behavior of CNDs; a physicochemical property of CNDs that influences their photocatalytic performance. Another important aspect to consider for their improvement is how other components of photocatalytic systems, such as electron donors (EDs) and molecular cocatalysts, influence the charge-transfer behavior of photoexcited CNDs.^19,20^ These interactions are pivotal to photocatalytic activity and can be tracked via spectroscopic techniques offering sufficient temporal and spectral range, in addition to sensitivity.^21,22^ Despite the information gleaned from previous work in this field, there is still much to be learnt about the physicochemical properties of CNDs, e.g. the fundamental origin of CND photoluminescence is a still a topic of debate, and how interactions between other components in photocatalytic systems influence their performance in photocatalytic systems. In part, this is due to prior investigations lacking sensitivity to dynamic processes through the 5 ns to 10 μs temporal domain.^9,20^

To uncover the physical mechanisms governing behavior in CND-based photochemistry, TA spectroscopy has been performed in this study in the subpicosecond to millisecond range together with steady-state and time-resolved PL spectroscopy to investigate the electron transfer dynamics governing the photocatalytic behavior of different CND-ED combinations in the presence of methyl viologen (MV^2+^). MV^2+^ was chosen owing to its suitability as an indicator for electron transfer processes due to the distinct visible absorption signature it displays upon forming a reversible, singly charged radical cation upon reduction (MV^•+^), in addition to its utilization as an electron relay cocatalyst in classic photocatalytic systems.^23−25^ We examine the distinct CND-based charge transfer processes observed in the presence of different EDs and discuss the repercussions for high-performance applications in light of the potential experimental issues relevant to the study of these systems, including photodegradation and atmospheric effects. Here, we focus on nitrogen-doped graphitic CNDs (NgCNDs) as photosensitizers, as they display superior ultraviolet and visible light absorption compared with amorphous CNDs (aCNDs; see UV–vis spectra—Figure S1), together with superior photocatalytic activity relative to both aCNDS and graphitic CNDs without nitrogen doping (gCNDs).^20^

## Results and Discussion

**Figure 1:**
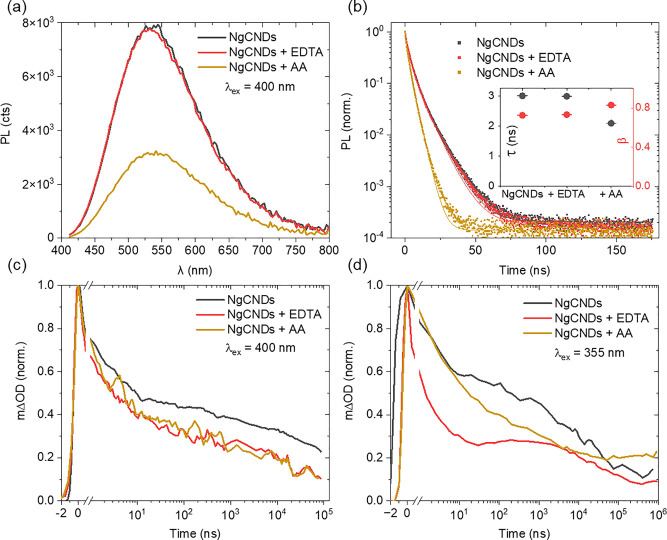
Optoelectronic characteristics of NgCNDs in aqueous solution (0.25 g L^–1^) with the sacrificial EDs EDTA and AA. (a) Photoluminescence spectra for NgCNDs (black), NgCNDs and EDTA (0.1 M, pH 6; red), and NgCNDs and AA (0.1 M, pH 4.5; orange) under continuous wave excitation (400 nm) at room temperature (298 K). (b) Normalized time-correlated single photon counting curves for the same sample configurations under pulsed excitation (404 nm). Solid lines are stretched exponential fits to the normalized kinetic (see Note S1). Inset: fitting parameters (τ and β) with associated error bars for each sample. (c) Long-time (ns–ms) spectrally averaged (530–770 nm) TA kinetics for NgCNDs in aqueous solution, and with added EDTA (pH 6) and AA (pH 4.5), under 400 nm pulsed excitation (see Materials and Methods). (d) Long-time spectrally averaged (530–770 nm) TA kinetics for NgCNDs in aqueous solution with added EDTA (pH 6) and AA (pH 4.5), under 355 nm pulsed excitation (see Materials and Methods). Note: Time before ‘0’ on the x-axis of (c) and (d) are on a standard, non-logarithmic scale, with all values >0 on a logarithmic scale.

Figure 1a shows the steady-state PL spectrum of NgCNDs in aqueous solution (0.25 g/L). Figure S2 shows the excitation dependence on the PL spectrum together with the emission dependence on the PL decay curves, with the maximum wavelength of PL observed to blue-shift as excitation energy is increased and eventually leading to little-to-no PL being observed at excitation wavelengths lower than 350 nm. This observation is common to CNDs and can be due to size-dependent optical selectivity of the NgCNDs and/or the presence of carboxylic acid functional groups on the surface of the NgCNDs introducing defects that act as nonemissive excitation energy traps.^26^ As we would expect these energy traps to only be accessed at certain excitation wavelengths, it follows that varying PL intensity would be observed relative to excitation wavelength, with excitation at 350 nm likely accessing a nonemissive excitation energy trap state due to the little-to-no PL observed at this excitation wavelength (Figure S2). Additionally, the introduction of the hole-scavenging molecules ethylenediaminetetraacetic acid (EDTA; 0.1 M, pH 8.0) and ascorbic acid (AA; 0.1 M, pH 4.5) has a different influence on the PL; EDTA causes no spectral or intensity changes to the dot PL, while AA causes significant PL quenching. This is most likely influenced by electrostatic interactions between the NgCNDs and EDs, whereby repulsive electrostatic interactions between NgCNDs and EDTA, both negatively charged at the pHs considered here, preclude any PL quenching. It is worth noting that the zeta potential of NgCNDs = −23 ± 1 mV from previous work,^20^ with zeta potential and hydrodynamic diameter generally decreasing as pH increases for carboxyl-terminated NgCNDs.^27^ EDTA presents in various negatively charged forms from ca. pH 2 upward, e.g. at pH 4.5, EDTA is almost exclusively in the double negatively charged state (i.e., EDTA^2–^); at pH 6, there is ca. a 1:1 ratio of EDTA^2–^ to EDTA^3–^.

However, as AA exists predominately in a singly charged form at pH 4.5 (i.e., less negatively charged than EDTA at this pH), there are less electrostatic repulsive interactions with NgCNDs and, as such, PL quenching via complexation of AA with NgCNDs becomes more favorable. This AA quenching is concomitant with faster decay of the time-resolved PL signal compared with EDTA and NgCND-only samples (Figure 1b). PL decay curves are fitted with a stretched exponential function reflecting the heterogeneity in transitions from which luminescence originates (see Note S1). EDTA has no appreciable influence on the characteristic decay parameter (τ) or heterogeneity parameter (β) but AA reduces τ by 30% and increases β by 14%. This increased quenching rate and reduction in “disorder” (increasing β) is suggestive of slower radiative transitions being preferably influenced by the introduction of AA.

To complement the nanosecond PL study, and before introducing any redox mediators, picosecond TA was used to confirm the ultrafast carrier dynamics and relaxation processes of the NgCNDs. Figure S3a,b show the three-dimensional TA data obtained from NgCNDs (0.25 g/L) dispersed in water at two different laser fluences (i.e., Figure S3a—14 μJ cm^–2^; Figure S3b—140 μJ cm^–2^), with water-only TA data shown for comparison (Figure S3c). In both cases, a characteristically broad photoinduced absorption (PIA) feature is exhibited across the full spectral range of our probe (510–775 nm—Figure S3a–e), decaying considerably over tens of picoseconds (Figure S3d). This positive transient is the signature of multiple optically allowed transitions from newly populated excited states to higher excited states of the NgCNDs and is consistent with that measured via steady-state UV–vis absorption spectroscopy.^22^ The photoinduced absorbance feature decays within the region 510–775 nm and approximately 7.5% of the initial signal intensity remains after 1 ns (Figure S3g). The decay kinetic of the NgCND absorbance feature within this range is also independent of excitation pulse fluence (Figure S3f), indicating it is governed by some first-order relaxation process within the fluence range investigated. A closer examination of the spectrally integrated kinetics reveals that the relaxation rate of the absorbance feature deviates slightly at the high energy edge of our probed spectral region (515–520 nm; see Figure S3h and Note S1 for fitting function).^20^ These distinct kinetics might indicate respective relaxation at bulk and surface states of the carbon dots. Surface states have been identified as facilitating PL decay in carbon dots,^18^ but the longer carrier lifetimes from PL measurements (cf. Figure 1)^28^ suggest that a proportion of the TA decay may be linked to fast carrier trapping or an alternative nonradiative relaxation process, with both phenomena attributed to CND bulk states.

At time scales of more relevance to charge transfer in photocatalytic applications through diffusion-controlled mass transport (> ns), the TA data shows distinct features which correlate with different physicochemical properties of the CNDs than those attributed to ultrafast relaxation processes (≪ ns). Figure 1c shows the long-time (pump–probe delay from ns to ms) spectrally averaged TA data for NgCNDs in an aqueous solution with and without the presence of EDs (i.e., EDTA and AA) excited by 400 nm pulses. The ultrafast relaxation is contained within the instrument response for this configuration (> 500 ps). In all cases, the rapid relaxation of the NgCNDs continues for 10 ns before a more gradual relaxation takes over, likely trap-mediated. The initial rapid decay in Figure 1c is likely related to the fluorescence decay times observed (cf. Figure 1b), with both displaying faster decay rates when EDs are present.

The addition of the EDs EDTA (pH 6) and AA (pH 4.5) have distinctly different effects on the relaxation of the CNDs, depending on pump excitation wavelength. With 400 nm excitation, both EDs slightly quench the PIA of the dots and reduce the lifetime of the latter decay, but little difference is observed between the EDTA and AA samples (Figures 1c and S4g,h). However, distinct differences are observed upon 355 nm excitation (Figure 1d). For the case of EDTA, a more rapid initial quenching of the CND absorption within 30 ns of excitation is followed by a rise in the PIA before the decay resumes at >3 μs. Conversely, NgCND relaxation is more delayed in the presence of AA for the first 20 ns, with subsequent faster quenching of the absorption feature. This difference in TA kinetics is most likely due to EDTA favoring interactions with nonradiative excited states of the NgCNDs that are accessed when excited at 355 nm rather than 400 nm (rationalized by the lack of PL intensity at an excitation wavelength of 350 nm—Figure S2), leading to efficient charge transfer complexation of EDTA with optically excited NgCNDs that manifests as the rise in PIA observed at ca. 30 ns in the NgCND + EDTA TA trace in Figure 1d.

EDTA is a reductive quencher,^20^ which will fill the photoexcited hole of the dot before any further charge transfer processes take place. This is verified by comparison of the reduction potentials of both EDTA and CNDs*, which ensures that reductive quenching of CNDs* by EDTA is favorable (*E*^EDTA/EDTA–^ = −0.57 V vs NHE; *E*^CND^*^/CND–^ = −0.55 V vs NHE).^29,30^ As time scales below 100 ns are short for electron transfer processes to occur from the EDs to the dots, the rise in PIA observed at ca. 30 ns in the presence of EDTA could be due to the formation of a charge transfer complex between the NgCNDs and EDTA. This could be facilitated by the presence of ED molecules (which are in large excess of the dot population) affecting trapping and recombination through electrostatic or van der Waals interactions perturbing the surface state energies—as may also be the case for AA, which appears to screen surface trapping on nanosecond time scales before contributing to quenching at later times. As such, the ns−μs quenching of the dot PIA can be linked to this electron donation, providing evidence that kinetics in this regime are related to photoexcited holes.^20^

**Figure 2:**
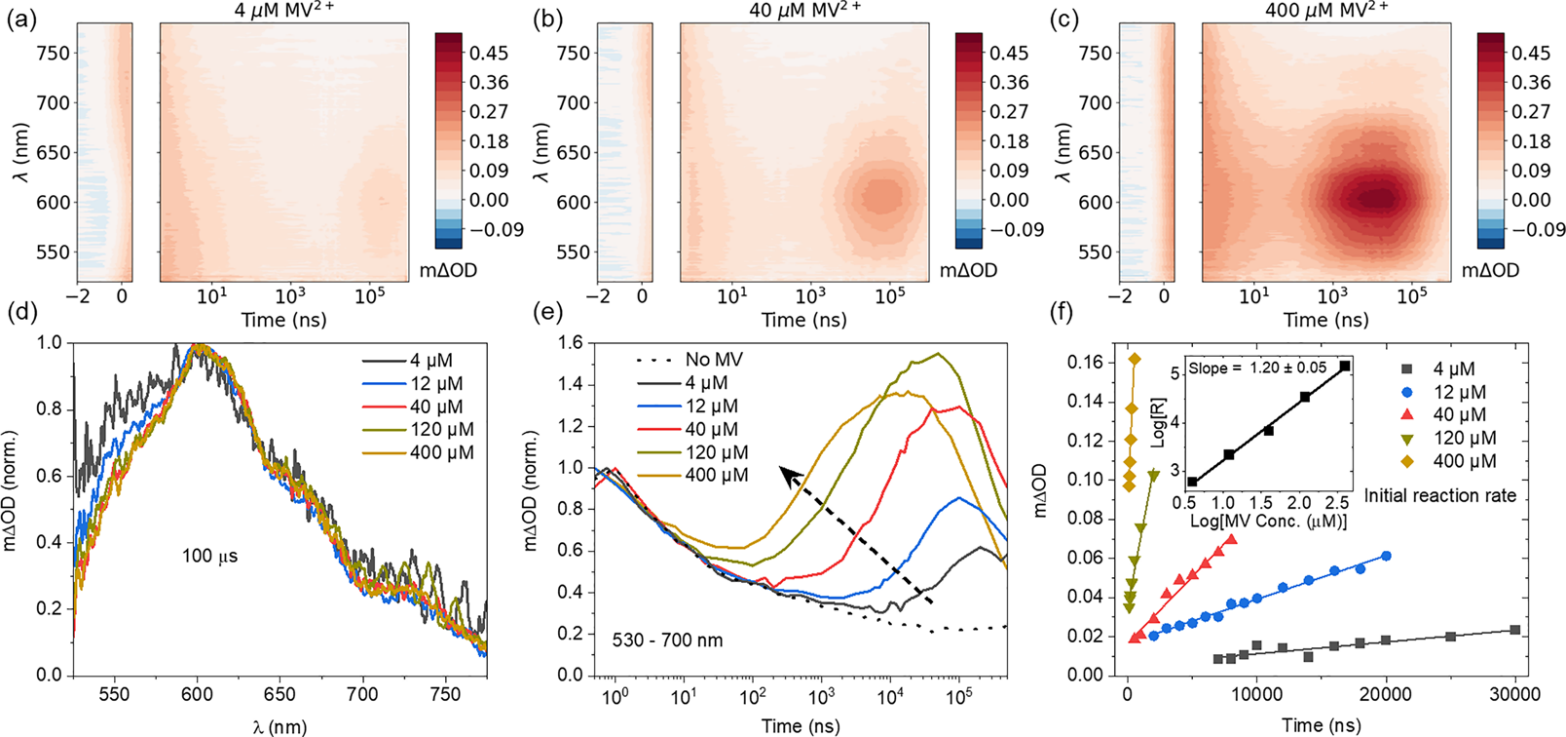
TA of the NgCND/AA/MV^2+^ system showing microsecond generation and quenching of MV^•+^ under 355 nm pulsed excitation. (a–c) Three-dimensional TA data for NgCNDs (0.25 g L^–1^) in aqueous solution with AA (0.1 M) and MV^2+^ added in a concentration series at the labeled molarities. (d) Normalized TA spectral slices extracted at a pump–probe delay of 100 μs, for varying MV^2+^ concentration. (e) Spectrally averaged (530–700 nm) kinetics for the MV^2+^ concentration series (normalized). The black dashed arrow indicates the trend of the MV^•+^ radical absorption feature toward earlier time and higher intensity. (f) Growth kinetics of the MV^•+^ radical absorption feature showing an increasing initial reaction rate with MV^2+^ concentration. Inset: the logarithm of the gradient (*R* = rate) extracted from each MV concentration series considered, plotted against the logarithm of the initial concentration of MV^2+^. This analysis using eq 2 reveals a first-order reaction rate dependence on MV^2+^ concentration.

As an indicator of charge transfer in photocatalytic systems owing to its distinctive absorption bands in the visible region, the methyl viologen dication (MV^2+^) was added in a concentration series to consistent concentrations of the dots (0.25 g/L) and AA (0.1 M) at pH 4.5 and the TA spectra subsequently recorded with 355 nm excitation of the NgCNDs. MV^2+^ has been studied previously in steady-state UV–vis investigations for electron transfer processes underlying CND-based photocatalytic systems used for hydrogen generation^21,22^ and as such, was deemed suitable for similar purposes for the TA experiments. Figure 2a–c show the ns–ms TA data whereby the MV^2+^ concentration is increased by an order of magnitude for each sample (see Figure S5a–c for intermediate concentrations). A new PIA feature is observed in the range 530–700 nm for all MV^2+^-containing samples (Figure 2d), rising between ∼300 ns (high concentration) and ∼30 μs (low concentration), and decaying toward the end of the millisecond time range (Figure 2e). The spectral shape of this new absorption feature, which peaks at 600 nm, is characteristic of the methyl viologen radical cation (MV^•+^) and is consistent across the concentration series (slice extract at pump–probe delay of 100 μs—see Figures 2d and S5c).^31,32^ The sub-100 ns relaxation of the NgCNDs appears unaffected by MV^2+^ concentration (see Figures 2e and S5d).

Oxidative quenching is a possible model for this radical cation formation, particularly since viologens are known to support oxidative quenching,^9,33^ together with the reduction potential of AA (which is primarily present in the singly charged form, HA^–^, at pH 4.5) as compared with that of CND*s making reductive quenching unfavorable (*E*^CND^*^/CND–^ = −0.55 V vs NHE; *E*^HA•/HA–^ = −0.46 V vs NHE).^29,30^ Specifically, a photoexcited dot directly transfers an electron to the MV^2+^ molecule before an AA molecule scavenges the remaining photoexcited hole. We expect this second process to occur faster than reductive quenching (as is the case for EDTA), which would be temporally limited by the initial ED hole scavenging.

Given the emergence of the MV^•+^ absorption band when AA is used as an ED, the kinetics of the concentration series can be examined, thus allowing for a better characterization of the NgCND-MV^2+^ interaction. In general, from Figure 2e we find both the onset and the peak time of the MV^•+^ PIA to shift to earlier pump–probe delay with increasing concentration of MV^2+^. The maximum signal of the PIA increases by a factor of 5 with a 100× increase in concentration (see Figures 2e and S5d). This will be influenced by the competition of reduction and relaxation, which also appears to increase in rate with concentration. The initial reduction reaction rate for MV^2+^ to MV^•+^ is extracted from the PIA onset, to confirm the reaction order (see Figure 2f). The reaction rate is a power law described as
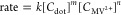
1where *C*_*i*_ is the concentration of the reactant *i*, *m* and *n* are the exponents dictating the order of each reactant, and *k* is the rate constant. Holding the dot concentration constant and taking the natural logarithm of eq 1 we determine the rate order of MV^2+^ (*n*) with the following linear relationship
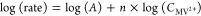
2where *A* is the constant term *k*[*C*_dot_]^*m*^. The slope extracted from Figure 2f fit is 1.20 ± 0.05, indicating the dependence of reaction rate on MV^2+^ concentration is approximately first order.

The decay of the MV^•+^ population on sub-ms time scales is inconsistent with the expected lifetime of these cations, whose population should build up under an average UV irradiance of ∼240 mW cm^–2^.^21^ The subsequent decay should span minutes when quenched by oxygen,^31^ or significantly longer time scales when shielded from air (as in our N_2_-purged setup).^32^ Diffusion of MV^•+^ out of the probed volume within 1 ms is precluded by its insufficient diffusivity in water.^34^

**Figure 3:**
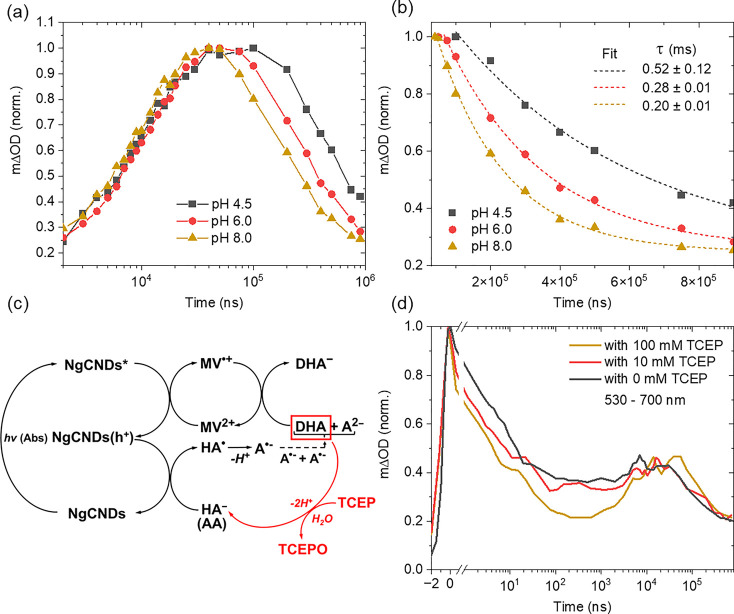
Investigating AA degradation as the primary influence on MV^•+^ quenching under 355 nm pulsed excitation of the NgCNDs. (a) Spectrally integrated (530–700 nm) TA kinetics of the NgCND (0.25 g L^–1^)/AA (0.1 M)/MV^2+^ (40 μM) system, at varying pH. Kinetics are normalized to the peak of the MV^•+^-related signal. (b) Decay kinetics of the MV^•+^-related signal. Dotted lines are fitted monoexponential decays. (c) Schematic of DHA formation during the AA oxidation cycle (starting with the singly deprotonated form of AA, i.e. HA^–^). When tris(2-carboxyethyl)phosphine (TCEP) is added to the photocatalytic system it acts as a SED-recycler by undergoing clean two-electron oxidation to form TCEPO, reducing DHA back to HA^–^/AA (indicated in red) in the process and allowing MV^•+^ to persist for longer pump–probe delays. (d) Spectrally integrated (530–700 nm) TA kinetics of the same configuration at pH 4.5 with the addition of TCEP (10 and 100 mM).

One explanation for the premature quenching could be that back electron transfer occurs from MV^•+^ to oxidized products of AA,^30,35,36^ quenching the PIA rapidly due to the 250–25,000× excess of AA in solution. Dehydroascorbic acid (DHA) is a product of AA oxidation, which is produced via two-electron and two-proton transfer (see Figure 3c).^37^ DHA formation is energetically more favorable under alkaline conditions where the initial H^+^ concentration is low.^38,39^ We find that the evolution of MV^•+^ radical is modified by the solution pH (see Figure 3a), with the formation of MV^•+^ confirmed by the identical spectral signatures shown in Figure S6. The onset and initial reaction rate are consistent, suggesting that these are only concentration-dependent. However, the quenching decay time of the radical cation population decreases from ∼500 μs at pH 4.5 to 200 μs at pH 8.0, suggesting the increased presence of DHA could trigger rapid oxidation of the MV^•+^ radicals (see Figure 3b). This behavior of quenching decay time as a function of pH is also likely reflective of the decreasing zeta potential and hydrodynamic diameter of the CNDs as pH increases,^27^ which would result in the oxidation of the CNDs* by MV^2+^ being less favorable and subsequently resulting in the decrease in the quenching decay time observed. The schematic Figure 3c illustrates the formation of DHA as part of the electron donation process to CNDs.

To explore the role of DHA as an oxidizing agent for MV^•+^, tris(2-carboxyethyl)phosphine (TCEP) is added to the NgCND-MV-AA solution (10, 100 mM). TCEP is a reducing agent which converts DHA back to AA, thus forming its oxidized product TCEPO, reversing the degradation pathway, and preventing the buildup of any significant DHA concentration.^30,40^ It is possible for TCEP to donate electrons directly to NgCNDs, but TCEP is far less efficient as an ED than AA, and that AA therefore dominates the donation of electrons to NgCNDs when TCEP is present.^30^ This is further validated by the TA measurements presented here, which show that the addition of TCEP leaves the TA kinetics qualitatively unchanged at time scales shorter than 10^3^ ns when election donation is expected to occur (see Figures 3d and S6). The appearance and decay of the radical cation signature occur on time scales similar to those of the systems employing only AA and 10 mM TCEP, the latter being present in a 0.1 molarity ratio to AA. However, when TCEP is added in a 1:1 molarity ratio with AA (which has yielded efficient performance in multiple photocatalytic systems^3,30,40^), there is a larger decrease in absorbance at 10^2^ – 10^3^ ns prior to the emergence of MV^•+^, which is observed to persist for slightly longer pump–probe delays than when TCEP is present in much lower concentrations (or not at all). This indicates that the addition of TCEP at a 1:1 molarity ratio with AA likely inhibits the formation of DHA via reduction by TCEP (Figure 3c), which further limits the potential quenching of MV^•+^ by DHA and allows the former to persist. The role of DHA in quenching MV^•+^ may be further rationalized with long-path length *in fibra* steady-state absorption measurements,^22^ which do reveal a difficulty in building up any MV^•+^ population over illumination periods of minutes when AA is used as the ED (Figure S7).

**Figure 4:**
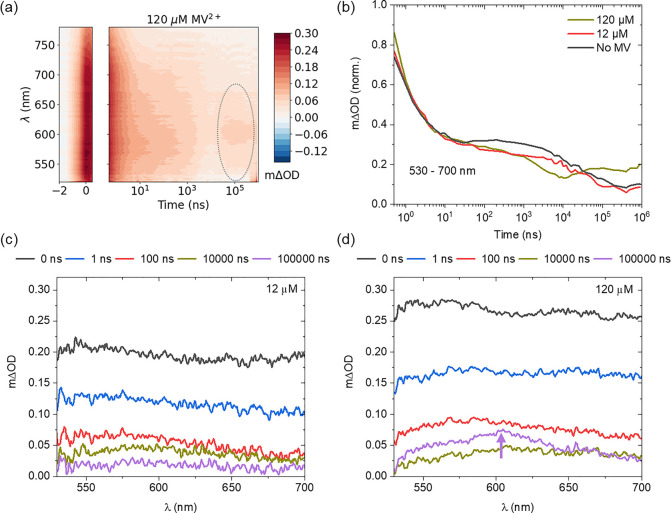
Slow EDTA-mediated charge transfer is accelerated at high methyl viologen concentration and pH 6 under 355 nm pulsed excitation of the NgCNDs. (a) 3D TA data for NgCND/EDTA/MV system with 120 μM MV^2+^ and 0.1 M EDTA (pH 6). Gray dotted region of interest marks the suspected presence of MV^•+^ radical PIA rise at >10 μs. (b) Comparison of spectrally averaged (530–700 nm) kinetics from selected samples from MV^2+^ concentration series with 0.1 M EDTA (pH 6). (c,d) TA spectra at several pump–probe delay times for system with (c) 12 and (d) 120 μM MV^2+^ with 0.1 M EDTA (pH 6). Purple arrow indicates the radical absorption peak in the characteristic wavelength range, at 100 μs.

Having explored the transient kinetics with AA as an ED, the transient kinetics with the same photocatalytic system but with a different ED, i.e. EDTA, were also investigated for comparative purposes. Figures 4 and S8 show the TA kinetics of an MV^2+^ concentration series in the NgCND-EDTA-MV^2+^ configuration. As with the AA configuration, the NgCND PIA dominates for up to 1 μs after excitation at 355 nm. At later times, the emergence of an MV^•+^ PIA signature is only weakly observed at high concentrations (see Figure 4a), contrary to the AA case. Figure 4b shows kinetic plots from samples with no MV^2+^, low concentration (12 μM), and high concentration (120 μM). The combination of EDTA and MV^2+^ appears to increase the quenching rate of the excited state dot, but only in the high-concentration sample is a subsequent MV^•+^ signal observed (indicated by the purple arrow in Figure 4d). Kinetic plots from all concentrations are shown in Figure S8. Low concentrations (MV^2+^ ≤ 40 μM) show minimal alteration from the sample without any MV^2+^. High concentrations (MV^2+^ ≥ 120 μM) see a drop in absorption in the 10^4^ ns range followed by a recovery in absorption at 10^5^ ns. This recovery is assigned to slower MV^•+^ radical formation than in the configuration with AA. Overall, these data fit with the picture of reductive quenching. The EDTA has a consistent effect on the TA kinetics at early time across each sample but no transfer or reduction of MV^2+^ is observed because this must occur on longer time scales, as is consistent with the reductive quenching of NgCNDs in the presence of EDTA and the DuBois-type catalyst NiP, i.e. EDTA reduces excited state CNDs on ns−μs time scales, with subsequent reduction of the DuBois-type catalyst by reduced CNDs on μs–ms time scales.^20^ Given that hole scavenging by EDTA happens in the ns−μs range^20^ (cf. Figure 1), NgCND-MV^2+^ interactions are likely screened by EDTA complexes forming with MV^2+^ and/or on the dot surface.^38,41^ In high MV^2+^ concentration samples, the 100 μs absorption rise at ∼600 nm suggests this screening might be surmountable within the 1 ms time range (see Figures 4b–d and S8). The absorption drop at 10 μs is thus linked to the initialization of the NgCND-MV^2+^ transfer.

A notable difference for the EDTA system is that rapid oxidation of any MV^•+^ is not expected other than via oxygen infiltration, meaning that the background (time-zero) population of MV^•+^ will build up over time, often over several minutes.^21,22^ TA kinetics for the NgCND-MV^2+^-EDTA system does depend on the measurement period in the extreme case of the sample being exposed for 10× the typical period (Figure S9). Long pump laser exposure smooths out the absorption profile relative to that of a fresh sample. In addition to establishing a background population of radicals, this level of exposure may trigger photobleaching of the methyl viologen, rendering it inactive within the photocatalytic system.^21^ This is less likely since methyl viologen is known to be stable through several photoluminescence and electroluminescence measurement cycles.^32^

**Figure 5:**
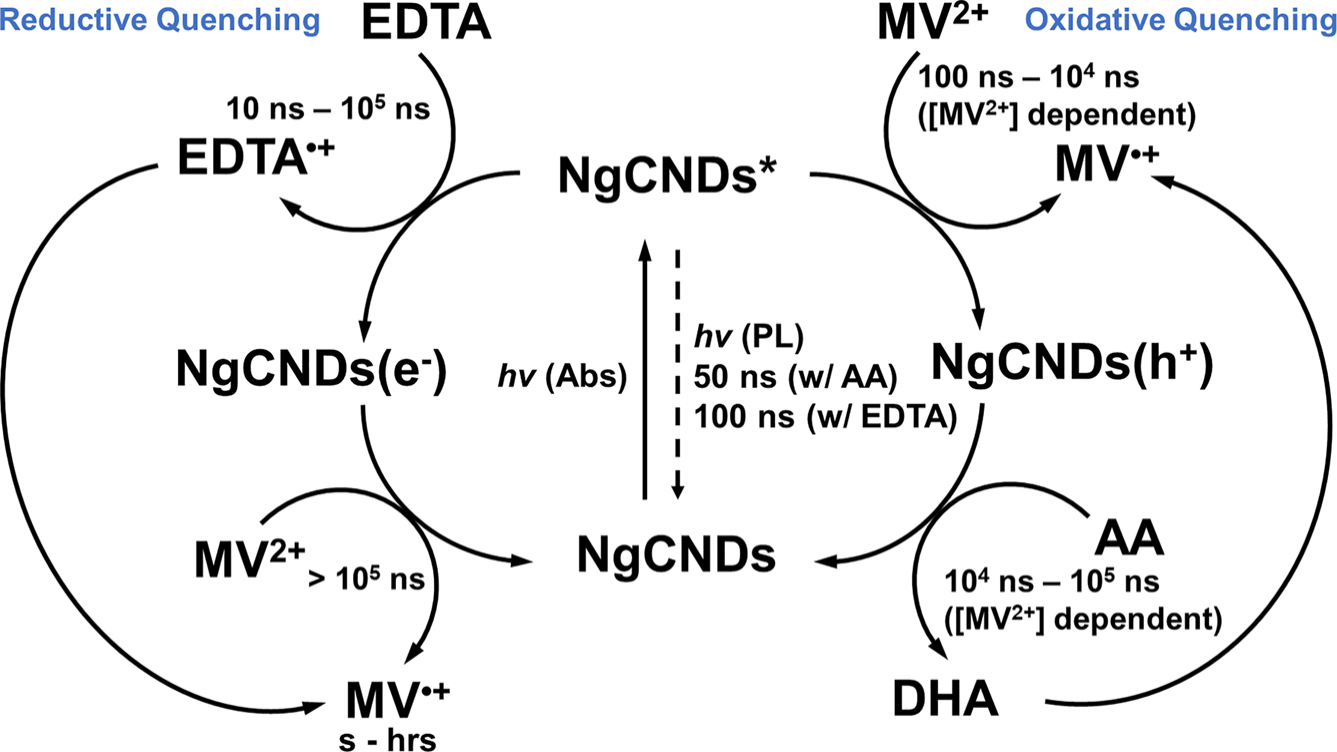
Representative diagram of the order and time scales of the various processes involved in NgCND-based photocatalytic systems relative to the sacrificial ED utilized. The overall process can be categorized as either reductive or oxidative quenching of the NgCNDs after initial photoexcitation.

The distinctive dynamics being observed between AA and EDTA in the TA spectra indicate that the choice of ED for a photocatalytic system is the primary influence for whether reductive or oxidative quenching of the NgCNDs occurs after initial photoexcitation (Figure 5). Upon 355 nm photoexcitation of NgCNDs in the presence of EDTA (with NgCNDs* lifetime of ca. 100 ns—Figure 1d and noted in Figure 5), the TA spectra indicate that the reductive quenching of NgCNDs* by EDTA to form NgCNDs(e^–^) via electron transfer occurs on a time scale approximately between 10 ns and 10^5^ ns (as noted in Figure 5), given that no difference in the kinetics is observed approximately below 10 ns for both EDTA and AA as SEDs (Figures 1c,d, 2e, 3d, and 4b), which is consistent with previously recorded TA spectra of NgCNDs in the presence of EDTA.^20^ The formation of NgCNDs(e^–^) is then subsequently followed by oxidative quenching of this species by MV to form MV^•+^ at >10^5^ ns after photoexcitation (Figure 4d and noted in Figure 5), which can then persist for seconds to hours as demonstrated by *in fibra* UV–vis steady-state measurements involving NgCNDs/EDTA/MV systems.^21,22^ The reductive quenching product, EDTA^•+^, and its decomposition byproducts are well-known to lead to radical decomposition of cocatalysts/electron relays in photocatalytic systems (as noted in Figure 5).^29,41^

In the presence of AA as an ED, and at the highest concentration of MV^2+^ (400 μM) and upon 355 nm photoexcitation of NgCNDs, the TA spectra start to show an increase in absorbance at 10^2^ ns most likely due to MV^•+^ formation (Figure 2e) resulting from the oxidative quenching of NgCNDs* (as noted in Figure 5). At the lowest concentration of MV^2+^ (4 μM), the onset of absorbance occurs at a pump–probe delay time of 10^4^ ns (Figure 2e), indicating that the time scale of reductive quenching of NgCNDs* by MV^2+^ is dependent on the initial concentration of MV^2+^ (as noted in Figure 5). As MV^2+^ concentration decreases, the MV^•+^ persists for longer pump–probe delays but the lifetime of the MV^•+^ signal stays approximately the same (Figure 2e). This indicates that a higher concentration of MV^2+^ leads to a significantly shorter diffusion time for MV^2+^ to interact with NgCNDs* and as such, more successful oxidative quenching collisions of NgCNDs* by MV^2+^ and/or a higher concentration of NgCND-MV^2+^ charge transfer complexes prior to electron transfer, resulting in the formation of MV^•+^ at earlier pump–probe delays. From here, the DHA resulting from interaction of NgCNDs(h^+^) with AA reacts with the MV^•+^ formed, taking approximately 100 ns at high initial MV^2+^ concentrations to significantly decrease the MV^•+^ population to baseline (i.e., time taken from the peak of the MV^•+^ signal to diminish in absorbance until baseline is reached in the TA spectra), with time scales and MV^2+^-concentration-dependence also noted in Figure 5. The reaction of DHA with MV^•+^ is further validated by the addition of TCEP reacting with DHA before it can react with MV^•+^, causing the MV^•+^ to appear at earlier pump–probe delays together with a higher absorbance and forcing the MV^•+^signal to persist for longer (Figure 3).

Overall, it appears that implementing AA as an ED with MV^2+^ as an electron relay not only affords oxidative quenching of NgCNDs* to form MV^•+^, but in the presence of TCEP allows for MV^•+^ to persist long enough within the relevant time window (i.e., 10^2^ – 10^5^ ns) to allow for effective electron transfer to a substrate either directly or via a catalyst (e.g., Colloidal Pt in more classical photocatalytic systems) to occur. However, as implementing EDTA as an ED begins with the reductive quenching of NgCNDs*, the resulting oxidation products of EDTA inevitably degrade electron relay/catalysts in photocatalytic systems (such as MV^•+^ here). Coupled with the observation that MV^•+^ formation via the quenching of NgCNDs(e^–^) by MV^2+^ occurs on time scales that are nonoptimal for efficient electron transfer processes to occur (>10^5^ ns), this could provide additional rationale for the observed reduction in photocatalytic H_2_ generation when EDTA is used as an ED in CND-photosensitized systems, e.g. CND-molecular nickel bis(diphosphine) photocatalyst systems–implementing TCEP/AA as the electron-donor system instead of EDTA with this photocatalyst system yielded record turnover numbers of 1094 ± 61 mol_H2_ (mol_Ni_)^−1^ with a benchmark lifetime of more than 5 days.^30^

## Conclusion

TA spectroscopy has been used to probe the excited state dynamics of NgCND-based photochemistry. Intriguingly, the choice of ED is determined to be crucial for dictating the reaction kinetics for a particular absorber and acceptor. This influence is not limited to directing a particular quenching mechanism (reductive or oxidative), but also highlights that complexes or degradation products of EDs can support or inhibit the buildup of intermediates, e.g. the MV^•+^ population. The NgCND-MV^2+^-AA photocatalytic system promotes the prompt formation of MV^•+^, most likely by oxidative quenching, but cannot maintain this population due to (detrimental) reoxidation by degradation products such as DHA. Meanwhile, the NgCND-MV^2+^-EDTA system does not see radical formation on sub-ms time scales due to an electrostatic screening effect, again attributable to the ED. However, this system is known to support longer-term radical formation. Future work will expand this investigation to include other EDs, such as triethylamine (TEA) and triethanolamine (TEOA), which are versatile and have been widely applied in configurations for photocatalysis.^29^ This study highlights the importance of understanding the formation of ED complexes and degradation products in the design of efficient photocatalytic systems.

## Materials and Methods

### Sample Preparation

All chemicals and reagents were purchased from commercial suppliers and used as received unless otherwise noted. Laboratory-grade reagents were used for synthesis, and chemicals for the analytical part were of the highest available purity. Phosphate buffer solutions (0.2 M) and ED stock solutions (0.2 M) were prepared and verified using a pH electrode (Mettler Toledo, FiveEasy Plus) at pH 6 and pH 8. The pH of ED stock solutions was adjusted using sodium hydroxide and hydrochloric acid. A methyl viologen (MV^2+^) stock solution was made up by dissolving methyl viologen dichloride. All stock solutions were made up using Milli-Q purified water.

NgCNDs were synthesized by pyrolysis of aspartic acid at 320 °C. All syntheses and characterization were carried out as previously reported.^20^ CNDs synthesized by these protocols give CND diameters of 3.1 nm for NgCNDs, 3.6 nm for gCNDs, and 6.8 nm for aCNDs.^20^

For all spectroscopic measurements, NgCNDs (0.25 g/L) were dispersed in aqueous solution. EDs EDTA (0.10–0.19 M) and AA (0.1–0.19 M) were added as detailed in the manuscript. For probing photocatalytic activity, the redox indicator methyl viologen (MV^2+^) was added in various molar volumes to alter the NgCND/MV^2+^ ratio in solution. Phosphate buffer was used with EDTA to maintain a constant pH.

Sample solutions were deposited under ambient conditions in a quartz cuvette (1 mm path length, Hellma), sealed with a screw cap. During TA measurements, samples were continuously purged with nitrogen to minimize the oxygen content within the cuvette. Samples were prepared directly before measuring unless specified (cf. degradation study).

### UV–vis Absorption

UV–vis absorption measurements were carried using a Cary 300 UV–vis spectrophotometer.

### Photoluminescence and Time-Correlated Single Photon Counting

Steady-state and time-resolved photoluminescence measurements were carried out using an Edinburgh Instruments FLS 1000 Photoluminescence Spectrometer.

### Transient Absorption Spectroscopy

Short-time (100 fs–2 ns) TA measurements were carried out using the second harmonic of a Ti/sapphire amplifier (Spectra-Physics Solstice) as the pump beam (90 fs pulse width, 500 Hz repetition rate, 3.1 eV photon energy). The probe spectrum was generated using a home-built noncollinear optical parametric amplifier, pumped by the second harmonic of the same Ti/sapphire amplifier. A motor-driven delay stage was used to mechanically vary the pump–probe delay.

Long-time (ns–ms) TA measurements were carried out in two different configurations.

- Configuration 1: the third harmonic of an electro-optical Q-switched laser (InnoLas picolo AOT) was used as the pump beam (< 800 ps pulse width, 500 Hz repetition rate, 3.49 eV photon energy). The probe spectrum was generated using a home-built noncollinear optical parametric amplifier, pumped by the second harmonic of the aforementioned Ti/sapphire amplifier. A delay generator was used to electronically vary the pump–probe delay.
- Configuration 2: the second harmonic of a Ti/sapphire amplifier (Spectra-Physics Solstice) was used as the pump beam (90 fs pulse width, 500 Hz repetition rate, 3.1 eV photon energy). The probe spectrum was generated by a broadband supercontinuum source (Leukos Disco). A delay generator was used to vary the pump–probe delay electronically.

## Data Availability

The data that support the findings of this study are openly available at the Apollo University of Cambridge data repository (https://doi.org/10.17863/CAM.110231).^42^

## Supporting Information Available

Notes, Figures and Tables and includes details on stretched exponential fittings, UV–vis absorption spectra of CNDs, additional photoluminescence spectra, short-time (fs–ns) TA data, additional long-time (ns−μs) TA data and steady-state UV–vis absorption data (PDF)

## References

[ref1] Sadjadi S. The Utility of Carbon Dots for Photocatalysis. In Emerging Carbon Materials for Catalysis; Sadjadi S., Ed.; Elsevier, 2021; pp 123–160, Chapter 4.

[ref2] Martindale B. C. M.; Hutton G. A. M.; Caputo C. A.; Reisner E. Solar Hydrogen Production Using Carbon Quantum Dots and a Molecular Nickel Catalyst. J. Am. Chem. Soc. 2015, 137 (18), 6018–6025. 10.1021/jacs.5b01650. 25864839

[ref3] Ladomenou K.; Landrou G.; Charalambidis G.; Nikoloudakis E.; Coutsolelos A. G. Carbon Dots for Photocatalytic H 2 Production in Aqueous Media with Molecular Co Catalysts. Sustain. Energy Fuels 2021, 5 (2), 449–458. 10.1039/D0SE01630F.

[ref4] Badiani V. M.; Casadevall C.; Miller M.; Cobb S. J.; Manuel R. R.; Pereira I. A. C.; Reisner E. Engineering Electro- and Photocatalytic Carbon Materials for CO2 Reduction by Formate Dehydrogenase. J. Am. Chem. Soc. 2022, 144 (31), 14207–14216. 10.1021/jacs.2c04529. 35900819 PMC9376922

[ref5] Rosso C.; Filippini G.; Prato M. Carbon Dots as Nano-Organocatalysts for Synthetic Applications. ACS Catal. 2020, 10 (15), 8090–8105. 10.1021/acscatal.0c01989.

[ref6] Cailotto S.; Negrato M.; Daniele S.; Luque R.; Selva M.; Amadio E.; Perosa A. Carbon Dots as Photocatalysts for Organic Synthesis: Metal-Free Methylene–Oxygen-Bond Photocleavage. Green Chem. 2020, 22 (4), 1145–1149. 10.1039/C9GC03811F.

[ref7] Han Y.; Huang H.; Zhang H.; Liu Y.; Han X.; Liu R.; Li H.; Kang Z. Carbon Quantum Dots with Photoenhanced Hydrogen-Bond Catalytic Activity in Aldol Condensations. ACS Catal. 2014, 4 (3), 781–787. 10.1021/cs401118x.

[ref8] Li H.; Sun C.; Ali M.; Zhou F.; Zhang X.; MacFarlane D. R. Sulfated Carbon Quantum Dots as Efficient Visible-Light Switchable Acid Catalysts for Room-Temperature Ring-Opening Reactions. Angew. Chem., Int. Ed. 2015, 54 (29), 8420–8424. 10.1002/anie.201501698. 26032183

[ref9] Achilleos D. S.; Yang W.; Kasap H.; Savateev A.; Markushyna Y.; Durrant J. R.; Reisner E. Solar Reforming of Biomass with Homogeneous Carbon Dots. Angew. Chem., Int. Ed. 2020, 59 (41), 18184–18188. 10.1002/anie.202008217. PMC7589312 33448554

[ref10] Lim S. Y.; Shen W.; Gao Z. Carbon Quantum Dots and Their Applications. Chem. Soc. Rev. 2015, 44 (1), 362–381. 10.1039/C4CS00269E. 25316556

[ref11] Liu Y.; Huang H.; Cao W.; Mao B.; Liu Y.; Kang Z. Advances in Carbon Dots: From the Perspective of Traditional Quantum Dots. Mater. Chem. Front. 2020, 4 (6), 1586–1613. 10.1039/D0QM00090F.

[ref12] D̵ord̵ević L.; Arcudi F.; Cacioppo M.; Prato M. A Multifunctional Chemical Toolbox to Engineer Carbon Dots for Biomedical and Energy Applications. Nat. Nanotechnol. 2022, 17 (2), 112–130. 10.1038/s41565-021-01051-7. 35173327

[ref13] Hutton G. A. M.; Reuillard B.; Martindale B. C. M.; Caputo C. A.; Lockwood C. W. J.; Butt J. N.; Reisner E. Carbon Dots as Versatile Photosensitizers for Solar-Driven Catalysis with Redox Enzymes. J. Am. Chem. Soc. 2016, 138 (51), 16722–16730. 10.1021/jacs.6b10146. 27977174

[ref14] Rosso C.; Filippini G.; Prato M. Use of Nitrogen-Doped Carbon Nanodots for the Photocatalytic Fluoroalkylation of Organic Compounds. Chem.—Eur. J. 2019, 25 (70), 16032–16036. 10.1002/chem.201903433. 31529711

[ref15] Wen X.; Yu P.; Toh Y.-R.; Hao X.; Tang J. Intrinsic and Extrinsic Fluorescence in Carbon Nanodots: Ultrafast Time-Resolved Fluorescence and Carrier Dynamics. Adv. Opt. Mater. 2013, 1 (2), 173–178. 10.1002/adom.201200046.

[ref16] Wang L.; Zhu S.-J.; Wang H.-Y.; Qu S.-N.; Zhang Y.-L.; Zhang J.-H.; Chen Q.-D.; Xu H.-L.; Han W.; Yang B.; Sun H.-B. Common Origin of Green Luminescence in Carbon Nanodots and Graphene Quantum Dots. ACS Nano 2014, 8 (3), 2541–2547. 10.1021/nn500368m. 24517361

[ref17] Strauss V.; Margraf J. T.; Dolle C.; Butz B.; Nacken T. J.; Walter J.; Bauer W.; Peukert W.; Spiecker E.; Clark T.; Guldi D. M. Carbon Nanodots: Toward a Comprehensive Understanding of Their Photoluminescence. J. Am. Chem. Soc. 2014, 136 (49), 17308–17316. 10.1021/ja510183c. 25372278

[ref18] Sui L.; Jin W.; Li S.; Liu D.; Jiang Y.; Chen A.; Liu H.; Shi Y.; Ding D.; Jin M. Ultrafast Carrier Dynamics of Carbon Nanodots in Different pH Environments. Phys. Chem. Chem. Phys. 2016, 18 (5), 3838–3845. 10.1039/C5CP07558K. 26763126

[ref19] Guttentag M.; Rodenberg A.; Kopelent R.; Probst B.; Buchwalder C.; Brandstätter M.; Hamm P.; Alberto R. Photocatalytic H2 Production with a Rhenium/Cobalt System in Water under Acidic Conditions. Eur. J. Inorg. Chem. 2012, 2012 (1), 59–64. 10.1002/ejic.201100883.

[ref20] Martindale B. C. M.; Hutton G. A. M.; Caputo C. A.; Prantl S.; Godin R.; Durrant J. R.; Reisner E. Enhancing Light Absorption and Charge Transfer Efficiency in Carbon Dots through Graphitization and Core Nitrogen Doping. Angew. Chem., Int. Ed. 2017, 56 (23), 6459–6463. 10.1002/anie.201700949. 28464489

[ref21] Koehler P.; Lawson T.; Neises J.; Willkomm J.; Martindale B. C. M.; Hutton G. A. M.; Antón-García D.; Lage A.; Gentleman A. S.; Frosz M. H.; Russell P. St. J.; Reisner E.; Euser T. G. Optofluidic Photonic Crystal Fiber Microreactors for In Situ Studies of Carbon Nanodot-Driven Photoreduction. Anal. Chem. 2021, 93 (2), 895–901. 10.1021/acs.analchem.0c03546. 33315379

[ref22] Lawson T.; Gentleman A. S.; Lage A.; Casadevall C.; Xiao J.; Petit T.; Frosz M. H.; Reisner E.; Euser T. G. Low-Volume Reaction Monitoring of Carbon Dot Light Absorbers in Optofluidic Microreactors. ACS Catal. 2023, 13 (13), 9090–9101. 10.1021/acscatal.3c02212. 37441232 PMC10334427

[ref23] Prasad D. R.; Hoffman M. Z. Photodynamics of the Tris(2,2’-Bipyrazine)Ruthenium(2+)/Methylviologen/EDTA System in Aqueous Solution. J. Am. Chem. Soc. 1986, 108 (10), 2568–2573. 10.1021/ja00270a013.

[ref24] Mandal K.; Hoffman M. Z. Quantum Yield of Formation of Methylviologen Radical Cation in the Photolysis of the Ru(Bpy)32+/Methylviologen/EDTA System. J. Phys. Chem. 1984, 88 (23), 5632–5639. 10.1021/j150667a035.

[ref25] Mulazzani Q. G.; Venturi M.; Hoffman M. Z. Radiolytically Induced One-Electron Reduction of Methylviologen in Aqueous Solution. Reactivity of EDTA Radicals toward Methylviologen. J. Phys. Chem. 1985, 89 (4), 722–728. 10.1021/j100250a032.

[ref26] Arsalani N.; Nezhad-Mokhtari P.; Jabbari E. Microwave-Assisted and One-Step Synthesis of PEG Passivated Fluorescent Carbon Dots from Gelatin as an Efficient Nanocarrier for Methotrexate Delivery. Artif. Cells Nanomedicine Biotechnol. 2019, 47 (1), 540–547. 10.1080/21691401.2018.1562460. 30829085

[ref27] da Silva A. O.; Rodrigues M. O.; Sousa M. H.; Campos A. F. C. pH-Dependent Surface Properties of N–Cdots Obtained by the Hydrothermal Method with Multicolored Emissions. Colloids Surf. Physicochem. Eng. Asp. 2021, 621, 126578 10.1016/j.colsurfa.2021.126578.

[ref28] Demchenko A. P.; Dekaliuk O. The origin of emissive states of carbon nanoparticles derived from ensemble-averaged and single-molecular studies. Nanoscale 2016, 8 (29), 14057–14069. 10.1039/C6NR02669A. 27399599

[ref29] Pellegrin Y.; Odobel F. Sacrificial Electron Donor Reagents for Solar Fuel Production. Compt. Rendus Chem. 2017, 20 (3), 283–295. 10.1016/j.crci.2015.11.026.

[ref30] Martindale B. C. M.; Joliat E.; Bachmann C.; Alberto R.; Reisner E. Clean Donor Oxidation Enhances the H 2 Evolution Activity of a Carbon Quantum Dot–Molecular Catalyst Photosystem. Angew. Chem., Int. Ed. 2016, 55 (32), 9402–9406. 10.1002/anie.201604355. 27355200

[ref31] Kong C.; Qin L.; Liu J.; Zhong X.; Zhu L.; Long Y.-T. Determination of Dissolved Oxygen Based on Photoinduced Electron Transfer from Quantum Dots to Methyl Viologen. Anal. Methods 2010, 2 (8), 1056–1062. 10.1039/c0ay00201a.

[ref32] McCune J. A.; Kuehnel M. F.; Reisner E.; Scherman O. A. Stimulus-Mediated Ultrastable Radical Formation. Chem. 2020, 6 (7), 1819–1830. 10.1016/j.chempr.2020.05.005.

[ref33] Narayanam J. M. R.; Stephenson C. R. J. Visible Light Photoredox Catalysis: Applications in Organic Synthesis. Chem. Soc. Rev. 2011, 40 (1), 102–113. 10.1039/B913880N. 20532341

[ref34] Holz M.; Heil S. R.; Sacco A. Temperature-Dependent Self-Diffusion Coefficients of Water and Six Selected Molecular Liquids for Calibration in Accurate 1H NMR PFG Measurements. Phys. Chem. Chem. Phys. 2000, 2 (20), 4740–4742. 10.1039/b005319h.

[ref35] Yuan J.-P.; Chen F. Degradation of Ascorbic Acid in Aqueous Solution. J. Agric. Food Chem. 1998, 46 (12), 5078–5082. 10.1021/jf9805404.

[ref36] Deutsch J. C. Dehydroascorbic Acid. J. Chromatogr. A 2000, 881 (1–2), 299–307. 10.1016/S0021-9673(00)00166-7. 10905713

[ref37] Tu Y.-J.; Njus D.; Schlegel H. B. A theoretical study of ascorbic acid oxidation and **HOȮ**/**O**_2_˙^–^radical scavenging. Org. Biomol. Chem. 2017, 15 (20), 4417–4431. 10.1039/C7OB00791D. 28485446

[ref38] Hoffman M. Z.; Prasad D. R.; Jones G.; Malba V. Formation of Photoactive Charge-Transfer Complexes between Methyl Viologen and Sacrifical Electron Donors. EDTA and Triethanolamine. J. Am. Chem. Soc. 1983, 105 (20), 6360–6362. 10.1021/ja00358a055.

[ref39] Blacker A. J.; Jazwinski J.; Lehn J.-M. Molecular Anion Binding and Substrate Photooxidation in Visible Light by 2,7-Diazapyrenium Cations. Helv. Chim. Acta 1987, 70 (1), 1–12. 10.1002/hlca.19870700102.

[ref40] Queyriaux N.; Giannoudis E.; Windle C. D.; Roy S.; Pécaut J.; Coutsolelos A. G.; Artero V.; Chavarot-Kerlidou M. A Noble Metal-Free Photocatalytic System Based on a Novel Cobalt Tetrapyridyl Catalyst for Hydrogen Production in Fully Aqueous Medium. Sustain. Energy Fuels 2018, 2 (3), 553–557. 10.1039/C7SE00428A.

[ref41] Prasad D. R.; Hoffman M. Z. Charge-Transfer Complexation between Methyl Viologen and Sacrificial Electron Donors EDTA, Triethanolamine, and Cysteine. J. Phys. Chem. 1984, 88 (23), 5660–5665. 10.1021/j150667a041.

[ref42] Macpherson S.; Lawson T.; Abfalterer A.; Andrich P.; Lage A.; Reisner E.; Euser T. G.; Stranks S. D.; Gentleman A. S. Research data supporting ‘The Influence of Electron Donors on the Charge Transfer Dynamics of Carbon Nanodots in Photocatalytic Systems’. Apollo – University of Cambridge, 2024. 10.17863/CAM.110231. PMC11334169 39169903

