# Supplementary material for: Influence of Electron Donors on the Charge Transfer Dynamics of Carbon Nanodots in Photocatalytic Systems
Source: ACS Catal. 2024 Jul 26;14(16):12006–15. doi: 10.1021/acscatal.4c02327 (PMC11334169; doi:10.1021/acscatal.4c02327)
Supplement: Supplementary file 1 — cs4c02327_si_001.pdf [file cs4c02327_si_001.pdf]

Supplementary Information for

The Influence of Electron Donors on the Charge Transfer Dynamics of Carbon Nanodots in Photocatalytic Systems

Stuart Macpherson[†1], Takashi Lawson[†1-3] Anna Abfalterer[1], Paolo Andrich[1], Ava Lage[3], Erwin Reisner[3], Tijmen G. Euser*[1], Samuel D. Stranks*[1,4] & Alexander S. Gentleman*[1,3]

Author affiliations:

1. Department of Physics, Cavendish Laboratory, University of Cambridge, Cambridge, CB3 0HE, United Kingdom
2. Department of Materials Science and Metallurgy, University of Cambridge, Cambridge, CB3 0FS, United Kingdom
3. Yusuf Hamied Department of Chemistry, University of Cambridge, Cambridge, CB2 1EW, United Kingdom
4. Department of Chemical Engineering and Biotechnology, University of Cambridge, Cambridge, CB3 0AS, United Kingdom

† These authors contributed equally

Corresponding authors: asg63@cam.ac.uk, sds65@cam.ac.uk, te287@cam.ac.uk

This PDF file includes:

Supplementary Note 1
Supplementary Figures 1-9
Supplementary References

Supplementary Note 1: On the use of stretched exponential fitting for carbon nanodot excited state kinetics.

Data in Figures 1b and S3h employ the model of a stretched exponential (equation S1) to describe the physical decay processes of nitrogen-doped graphitic carbon nanodots (CNDs). The stretched exponential intensity decay function contains two main parameters, the characteristic timescale of the decay (τ) and heterogeneity parameter (β), and takes the form:

$$I(t) = I_0 e^{-\left(\frac{t}{\tau}\right)^{\beta}}$$

where I_0 is the initial luminescence intensity. The β parameter is typically constrained to the range $0 < \beta < 1$, with β approaching 1 representing a homogeneous system obeying a single exponential decay, and β approaching 0 denoting a heterogeneous distribution of decays of varied timescales.

In the context of excited state relaxation in semiconductors, such a physical model is typically reserved for heterogeneous systems of species whose individual relaxation kinetics are first order but not singular (i.e., adopt a value within some range), and as a sufficiently disordered ensemble can be represented by this modified relationship.[1] This is particularly appropriate for nanoparticles with varying structural characteristics,[2] samples where long-range energy transfer governs luminescence kinetics,[3] or systems where local conditions vary spatially and/or temporally for emitters (e.g. in solution or biological samples),[4] leading to a distribution of decay constants for one physical system.

Studies of CNDs have frequently used this stretched exponential relationship to describe their decay kinetics, prompted by the inability to fit with single exponentials or justifiably with discrete multiexponential fits. The exact physical mechanism behind the heterogeneous decay behavior of CNDs is uncertain but possible explanations include variation in CND structure[5,6] or diverse surface-bulk state interactions across CNDs or between neighbours.[7,8] In terms of the information gathered from fitting with a stretched exponential, the characteristic timescale of decay can still be used as an indicator of the approximate quenching timescale of the dots emissive states, while the heterogeneity parameter reveals the diversity of such states that exist within a given sample.

1. Pelant, I. & Valenta, J. Kinetic description of luminescence processes. in *Luminescence Spectroscopy of Semiconductors* (Oxford University Press, 2012).
2. Zatryb, G., Podhorodecki, A., Misiewicz, J., Cardin, J. & Gourbilleau, F. On the nature of the stretched exponential photoluminescence decay for silicon nanocrystals. *Nanoscale Res. Lett.* **6**, 106 (2011).
3. Bodunov, E. N., Antonov, Yu. A. & Simões Gamboa, A. L. On the origin of stretched exponential (Kohlrausch) relaxation kinetics in the room temperature luminescence decay of colloidal quantum dots. *J. Chem. Phys.* **146**, 114102 (2017).
4. Lee, K. C. B. *et al.* Application of the Stretched Exponential Function to Fluorescence Lifetime Imaging. *Biophys. J.* **81**, 1265–1274 (2001).
5. Demchenko, A. P. & Dekaliuk, M. O. The origin of emissive states of carbon nanoparticles derived from ensemble-averaged and single-molecular studies. *Nanoscale* **8**, 14057–14069 (2016).
6. Sciortino, A., Pecorella, R., Cannas, M. & Messina, F. Effect of Halogen Ions on the Photocycle of Fluorescent Carbon Nanodots. *C* **5**, 64 (2019).

7. Terracina, A. *et al.* Photobleaching and Recovery Kinetics of a Palette of Carbon Nanodots Probed by In Situ Optical Spectroscopy. *ACS Appl. Mater. Interfaces* **14**, 36038–36051 (2022).
8. Jiang, Z. C. *et al.* A Facile and Low-Cost Method to Enhance the Internal Quantum Yield and External Light-Extraction Efficiency for Flexible Light-Emitting Carbon-Dot Films. *Sci. Rep.* **6**, 19991 (2016).

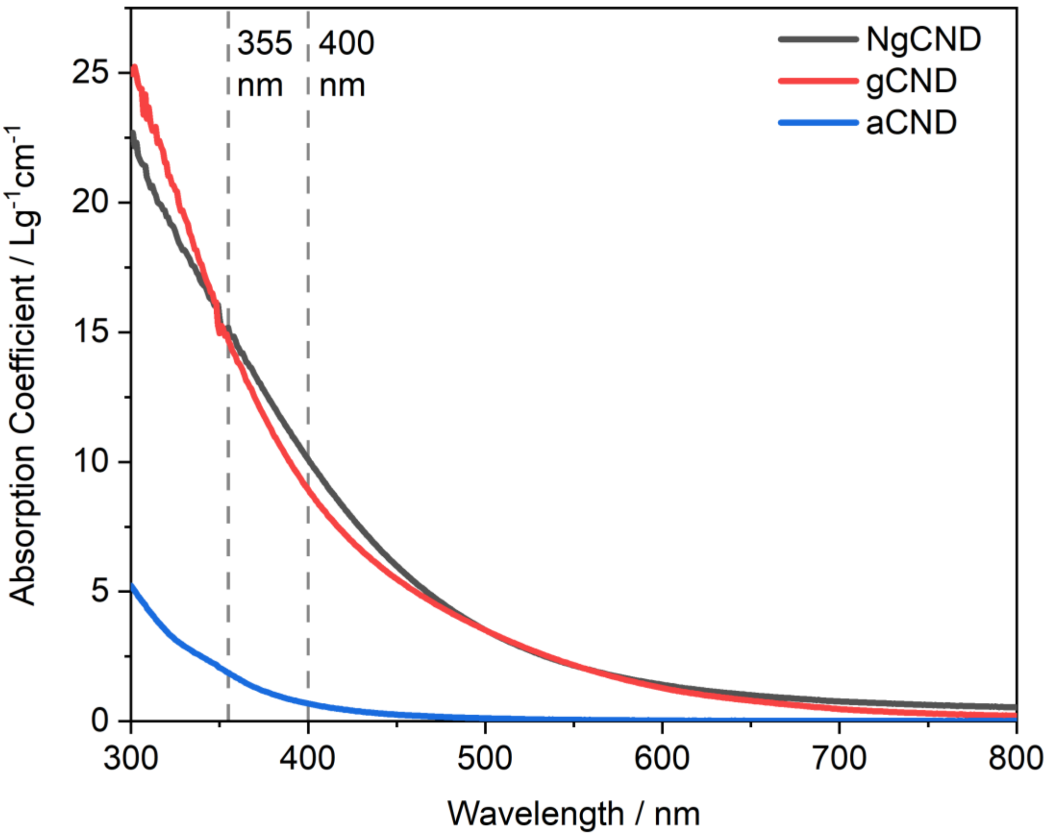

Figure S1: UV-Vis absorption of amorphous carbon nanodots (aCNDs; blue), graphitic carbon nanodots (gCNDs; red) and nitrogen-doped graphitic carbon nanodots (NgCNDs; black), in aqueous solution. Pump laser wavelengths for transient absorption in Figure 1 are indicated by dashed lines (355 nm and 400 nm).

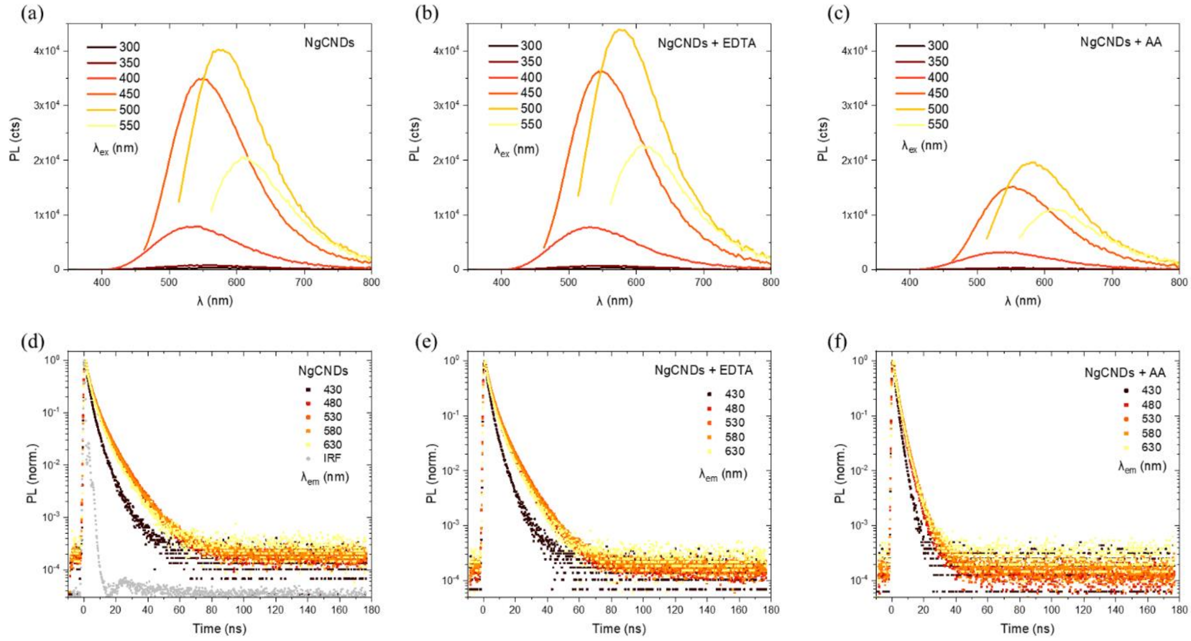

Figure S2: (a-c) Photoluminescence spectra under various steady-state **excitation** wavelengths (λ_{ex}), of (a) nitrogen-doped graphitic carbon nanodots (NgCNDs; 0.25 g L^{-1}), (b) NgCNDs (0.25 g L^{-1}) and ethylenediaminetetraacetic acid (EDTA; 0.1 M at pH 6), and (c) NgCNDs (0.25 g L^{-1}) and ascorbic acid (AA; 0.1 M). (d-f) Photoluminescence decays of the same samples at various **emission** wavelengths (λ_{em}) acquired using time-correlated single photon counting with pulsed excitation (404 nm, 5 MHz repetition rate, 3 nJ cm^{-2} $pulse^{-1}$). The instrument response function (IRF) is plotted as the grey trace in panel d.

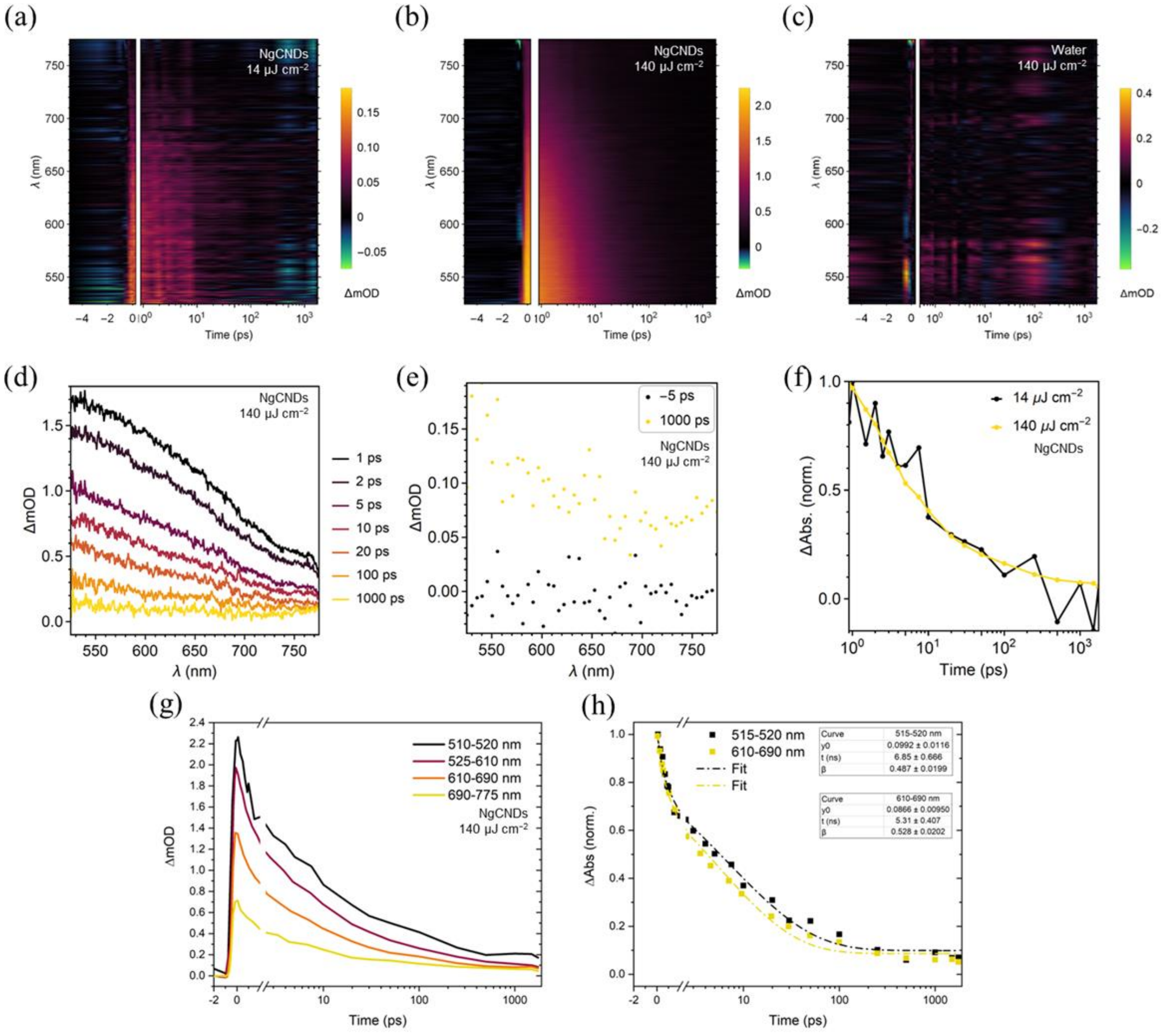

Figure S3: Short-time (fs-ns) transient absorption (TA) data for nitrogen-doped graphitic carbon nanodots (NgCNDs; 0.25 g/l in aqueous solution). (a-c) Chirp-corrected TA datasets with a partially logarithmic time axis, for NgCNDs with 400 nm pump excitation at fluence (a) 14 µJ cm^{-2} $pulse^{-1}$ and (b) 140 µJ cm^{-2} $pulse^{-1}$. (c) TA dataset for aqueous reference sample (Milli-Q water); 400 nm pump excitation at 140 µJ cm^{-2} $pulse^{-1}$. (d) Spectral slices from the TA dataset of NgCNDs under 400 nm, 140 µJ cm^{-2} $pulse^{-1}$ excitation. (e) Comparison of TA signal before excitation (−5 ps) and at a pump-probe delay of 1000 ps. Approximately 5% of the maximum photoinduced absorbance remains at 1000 ps. (f) Comparison of spectrally averaged (525-775 nm) kinetics for NgCNDs at different fluences from panels a and b. **(g)** Comparison of spectrally-averaged kinetics for NgCNDs (fluence = 140 µJ cm^{-2} $pulse^{-1}$). **(h)** Spectrally averaged high (515-520 nm) and low (610-690 nm) energy kinetics (fluence = 140 µJ cm^{-2} $pulse^{-1}$), each fitted with a stretched exponential (dashed line; see methods). Inset: Time constant, t, and stretch parameter, β, for each curve.

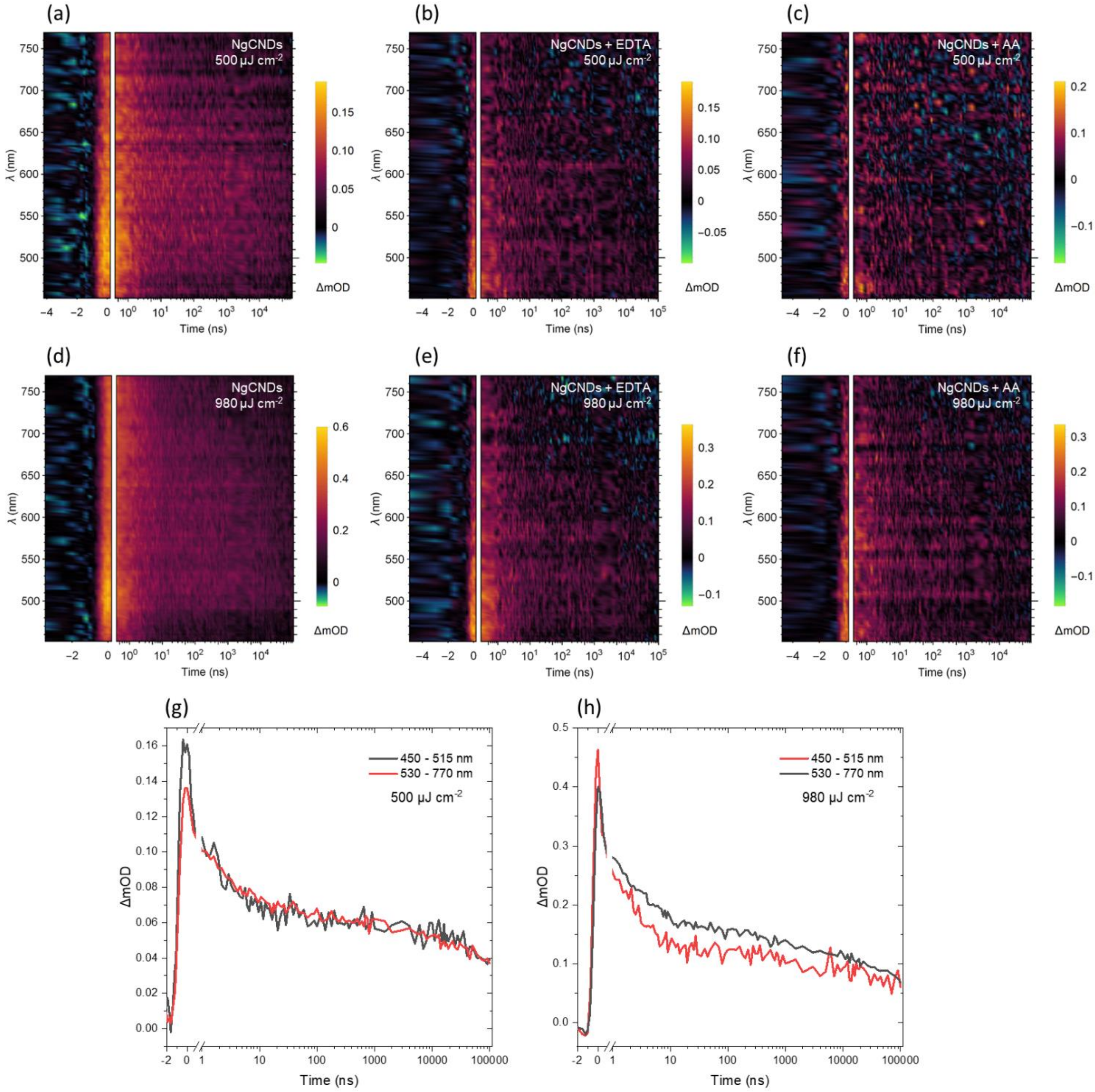

Figure S4: Additional long-time transient absorption data (pump excitation at 400 nm) for nitrogen-doped graphitic carbon nanodots in aqueous solution (0.25 g L^{-1}), and with added electron donors (EDTA, AA; both 0.1 M). (a-c) Three-dimensional TA data (500 μJ cm^{-2} $pulse^{-1}$) for NgCNDs alone and with added EDs. (d-f) Three-dimensional TA data (980 μJ cm^{-2} $pulse^{-1}$) for NgCNDs alone and with added EDs. (g,h) Spectrally integrated TA kinetics for NgCNDs at two different excitation fluences. Higher fluence excitation appears to slightly increase the overall decay rate, particularly at higher energies.

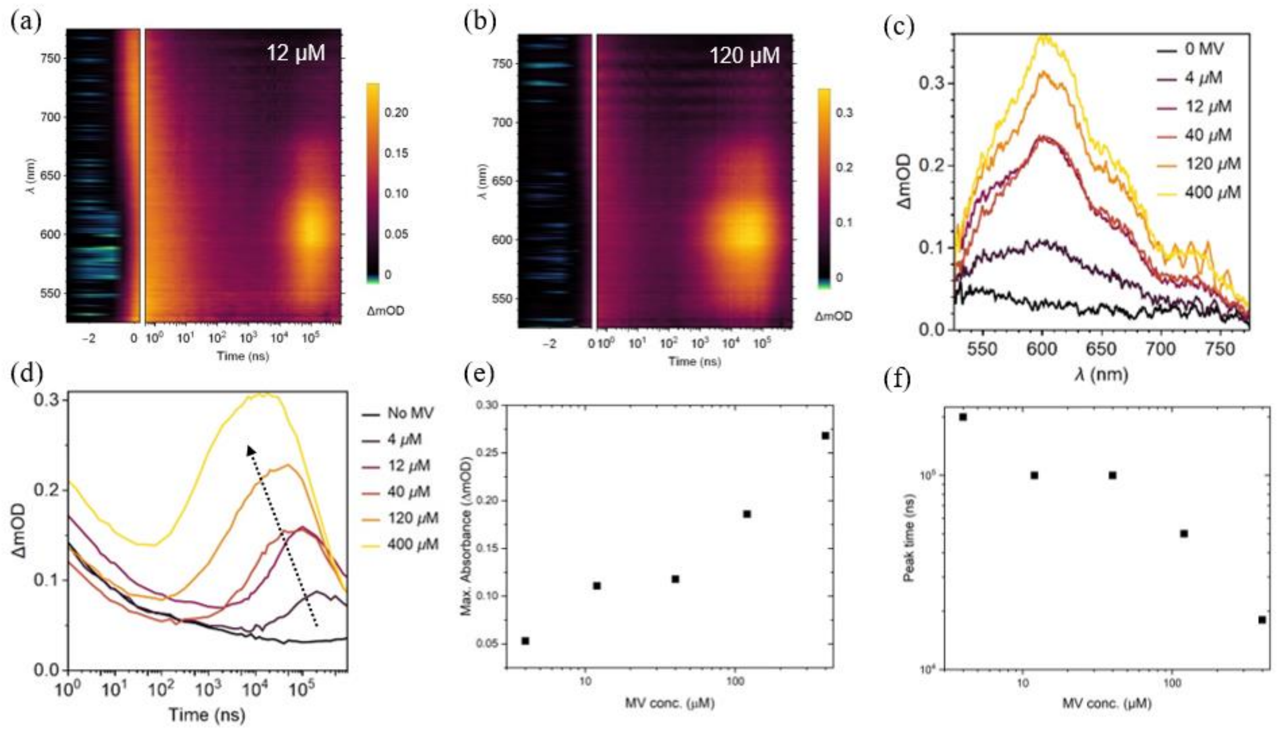

Figure S5: Additional long-time TA data for nitrogen-doped graphitic carbon dot / ascorbic acid / methyl viologen system. (a,b) 3D TA data for NgCND (0.25 g L^{-1}), AA (0.1 M) and MV^{2+} added with (a) 12 µM and (b) 120 µM molarity. (c) TA spectral slices extracted at a pump-probe delay of 100 µs, for varying MV^{2+} concentration. (d) Spectrally averaged (530 – 700 nm) kinetics for the MV^{2+} concentration series. The black dashed arrow indicates the trend of the onset of the $MV^{\cdot+}$ radical absorption towards earlier time. (e) Peak absorbance signal of the $MV^{\cdot+}$ related signal, compared by concentration. (f) Delay time at which the peak absorbance of the $MV^{\cdot+}$ related signal occurs, compared by concentration.

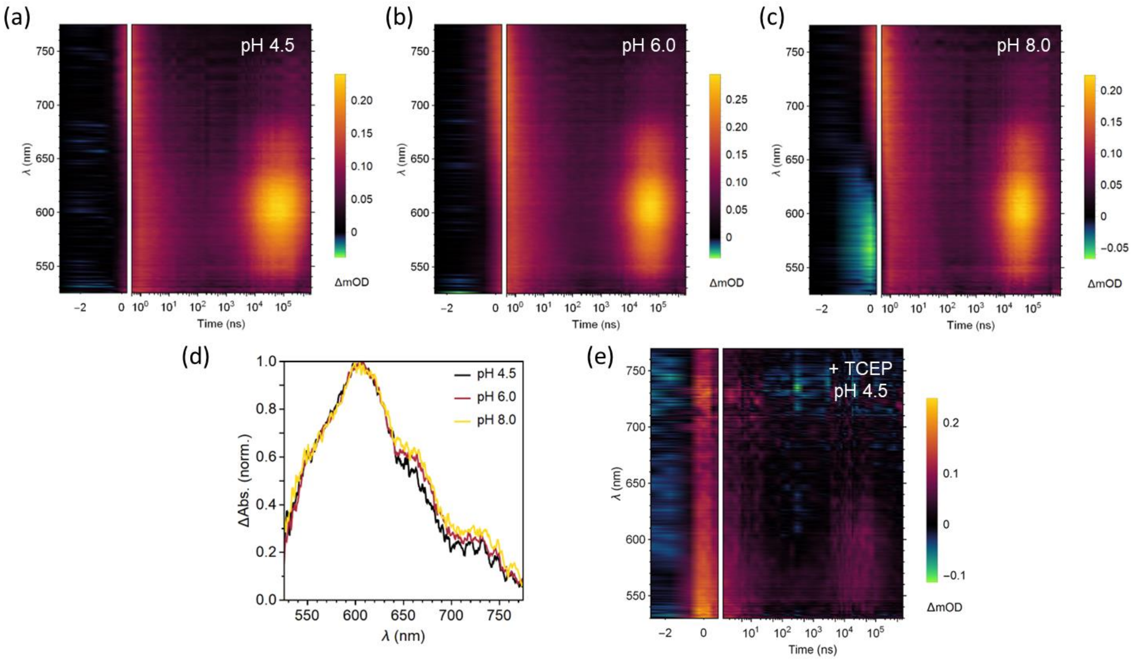

Figure S6: Long-time (ns-ms) transient absorption data for nitrogen-doped graphitic carbon nanodots (0.25 g/l) in aqueous solution with ascorbic acid (0.1 M) and methyl viologen (40 µM), at (a) pH 4.5, (b) pH 6.0, and (c) pH 8.0. (d) Normalized TA spectral slices extracted at a pump-probe delay of 50 µs, for varying sample pH. (e) Long-time (ns-ms) transient absorption data for nitrogen-doped graphitic carbon nanodots (0.25 g/l) in aqueous solution with ascorbic acid (0.1 M), tris(2-carboxyethyl)phosphene (0.1 M) and methyl viologen (40 µM), at pH 4.5.

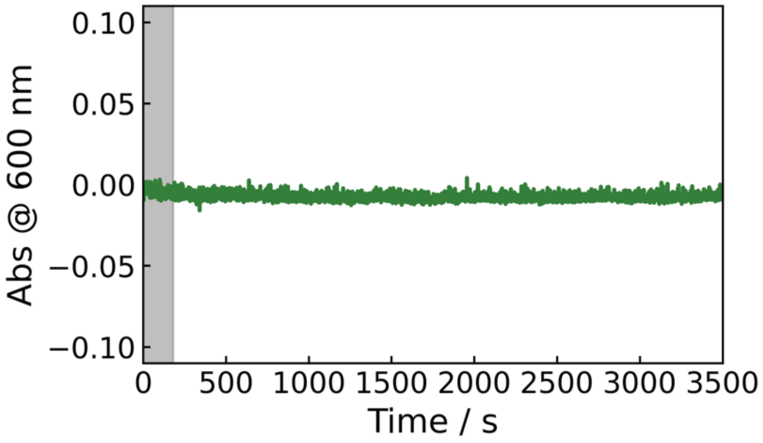

Figure S7: Monitoring the emergence of $MV^{•+}$ of a NgCND / AA / MV system with 40 µM MV^{2+} infiltrated within a Kagome-style hollow-core photonic crystal fiber (HC-PCF) – See Reference 22 in manuscript for further details regarding the experimental setup. The photoreduction process was initiated via external irradiation (λ_{irr} = 355 nm) of the HC-PCF. Unlike when EDTA is utilized as a SED, no trace of $MV^{•+}$ was observed to form during irradiation when AA is the SED.

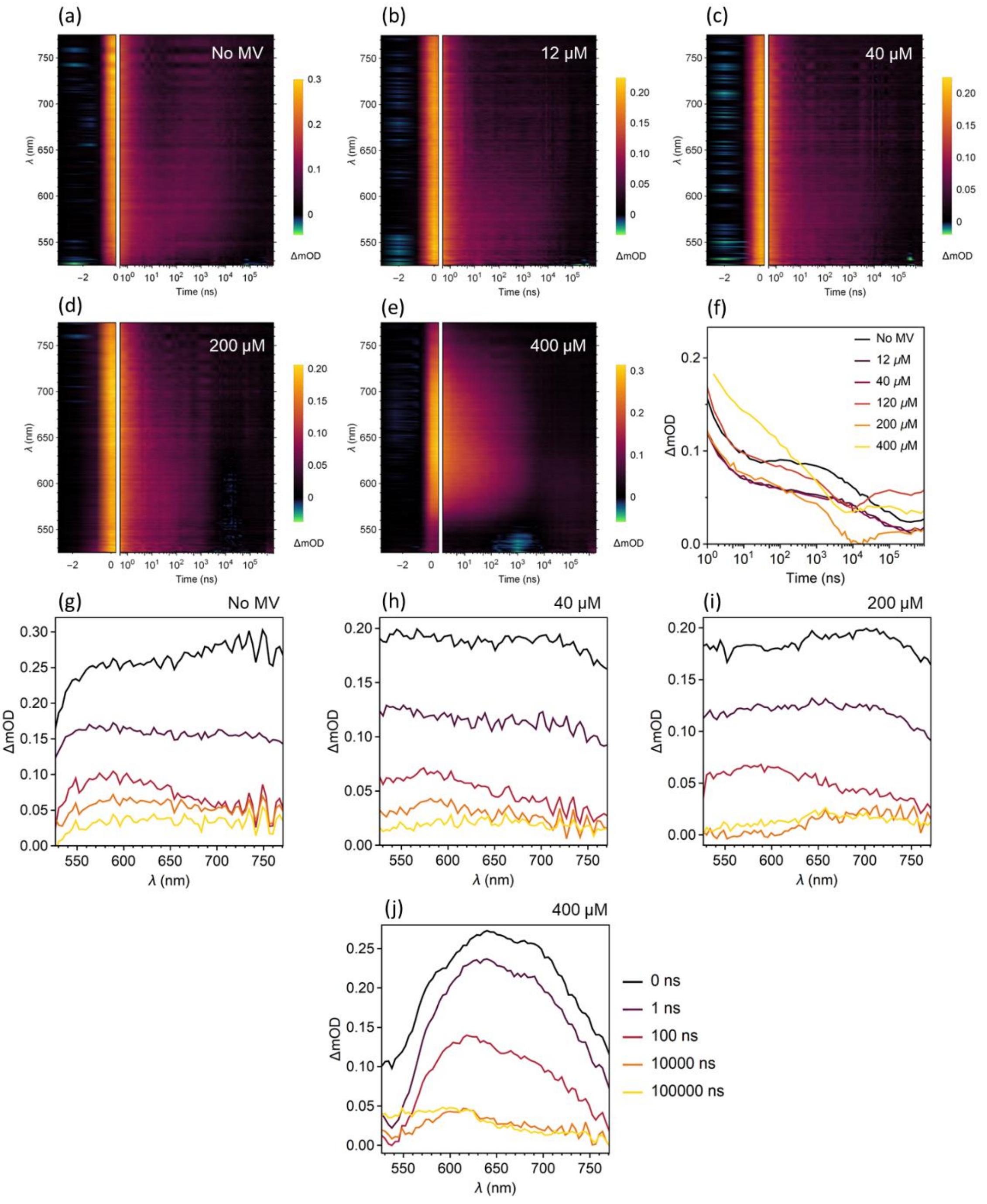

Figure S8: Additional long-time transient absorption data for nitrogen-doped graphitic carbon dot (0.25 gL^{-1}) / ethylenediaminetetraacetic acid (0.1 M) / methyl viologen system, at pH 6 unless stated. (a-e) Three-dimensional TA data for remaining MV^{2+} concentration series. (f) Spectrally integrated (550 – 650 nm) TA kinetics for full MV^{2+} concentration series. (g-j) Selected TA spectral slices for remaining MV^{2+} concentration series.

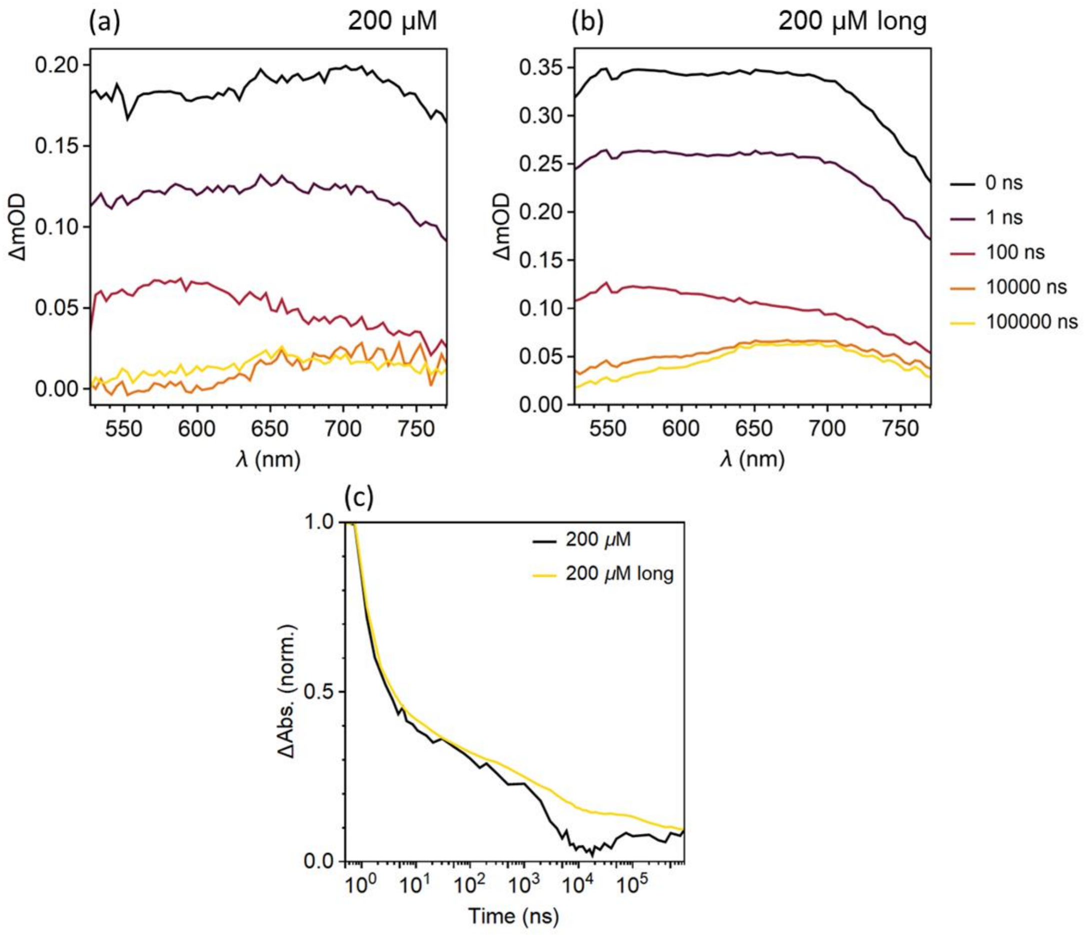

Figure S9: Irreversible chemical conversion of MV species. (a) Normalized TA spectra at several pump-probe delay times for a freshly prepared NgCND-MV^{2+} hybrid photocatalytic system with EDTA as a sacrificial electron donor. The MV^{2+} molarity is 200 µM, and the calculated ratio of MV^{2+} to dots is 5:1. Data was acquired for 26 measurement sweeps (~50 minutes). (b) Normalized TA spectra at several pump-probe delay times for the same sample after 260 measurement sweeps (~8 hours). At pump-probe delays < 1000 ns, only the signal:noise ratio is improved, but at delays of 10,000 and 100,000 ns there is a change in spectral shape in the wavelength range 530-650 nm. (c) Comparison of spectrally averaged (530-760 nm) kinetics for the short and long exposure samples.
